# Effect of a weight loss program on serum adiponectin and insulin resistance among overweight and obese premenopausal females

**DOI:** 10.1186/s42506-020-00060-z

**Published:** 2020-12-01

**Authors:** Walaa H. Foula, Rana H. Emara, Mona K. Eldeeb, Samiha A. Mokhtar, Fikrat A. El-Sahn

**Affiliations:** 1 Nutrition Department, High Institute of Public Health, Alexandria University, Alexandria, Egypt; 2 Chemical Pathology Department, Medical Research Institute, Alexandria University, Alexandria, Egypt; 3 Biostatistics Department, High Institute of Public Health, Alexandria University, Alexandria, Egypt

**Keywords:** Adipokines, Adiponectin, Insulin resistance, Weight reduction

## Abstract

**Background:**

Obesity has emerged as a public health crisis in many populations including Egypt. Adipose tissue produces a number of adipokines, one of them is adiponectin which has attracted much attention because of its antidiabetic and antiatherogenic effects.

**Objective:**

To determine the effect of a weight loss program on serum adiponectin level and insulin resistance among overweight and obese adult premenopausal females.

**Study design:**

A pre-postintervention study was carried out among 95 premenopausal overweight and obese females (body mass index ≥ 25 kg/m^2^) aged 20 to 40 years at the integrated health clinic affiliated to the High Institute of Public Health, Alexandria, Egypt, from February 2016 to February 2017. All participants underwent a weight loss program based on a reduced calorie balanced diet and advised to increase their physical activity. Dietary instructions and follow-up were done weekly throughout 16 weeks. Blood samples were collected to investigate serum adiponectin level and insulin resistance at the beginning and the end of the intervention.

**Results:**

After 16 weeks, a significant decrease in body weight by 9.7% was associated with a significant increase in serum adiponectin from 13.3 ± 4.9 μg/ml to 18.5 ± 5.6 μg/ml. Both fasting insulin and insulin resistance had decreased significantly by 13.6% and 13.7%, respectively.

**Conclusion:**

A weight reduction program depending on a reduced calorie diet for 16 weeks was associated with a significant increase in total adiponectin level and reduction in insulin resistance. An emphasis on the importance of keeping normal weight through nutritional education and the promotion of healthy diets is recommended to reduce the risk of occurrence of insulin resistance, type 2 diabetes, and cardiovascular diseases.

## Introduction

Obesity is considered now a major public health problem. In 2014, more than 1.9 billion adults all over the world (about 39%) were overweight. Of these, over 600 million (about 13%) were obese [1]. According to the results of the Egypt Demographic and Health Survey 2014, 36.5% of women aged 15–49 years were overweight and 48.1% were obese [2]. The risk of diabetes, hypertension, and dyslipidemia increases with obesity.

Adipose tissue is a highly active endocrine organ that produces a number of hormones and other substances called “adipokines” [3], such as tumor necrosis factor (TNF-α), interleukin-6 (IL-6), leptin, and adiponectin [4]. Adiponectin has a special role because of its antidiabetic and antiatherogenic effects [5]. Plasma adiponectin levels in humans range from 3 to 30 μg/ml [5]. It accounts for 0.01% of the total human plasma proteins, and this makes it the most abundant adipose tissue protein [6]. Plasma levels were significantly lower in men than women [7], obese subjects [8], metabolic syndrome [9] and type 2 diabetic patients [10], and those with coronary artery disease [10].

Caloric reduction is an easy and cost-effective measure in the management of obesity and comorbidities [11, 12]. Studies comparing adiponectin levels after weight loss programs are inconclusive [13–15]. The aim of the present study was to investigate the effect of a 16-week weight reduction program depending on a balanced low-calorie diet on serum total adiponectin level and insulin resistance among a group of overweight and obese premenopausal females.

## Methods

### Study design

One group pre-post intervention study design was carried out. The field study was conducted from February 2016 to February 2017.

### Study setting

The study was carried out in the integrated health clinic affiliated to the High Institute of Public Health (HIPH), Alexandria, Egypt.

### Participants and sample size

All subjects were premenopausal, nonpregnant, nonlactating overweight and obese (BMI ≥ 25 kg/m^2^) adult females aged 20 to 40 years. They showed no evidence of heart, renal, liver, cancer, diabetes mellitus, or any other medical disorders, or history of surgeries for obesity, or taking medications (oral steroids, thiazolidinediones, angiotensin-converting enzyme inhibitors, angiotensin II receptor blockers, clonidine like sympatoinhibitory antihypertensive agent and fenofibrate).

The study started by 122 overweight and obese adult females and ended by 95, and the dropout rate was 22%. A total sample of 95 overweight and obese adult females was required to estimate the effect size of the weight loss program for change at adiponectin level = 0.30, using alpha error = 0.05 and dropout rate = 10% and baseline adiponectin level = 2.98 ± 1.0 μg/ml [16]. This sample size was calculated using G.power software. Two days of the week were selected, and all subjects who attended the study setting and were fulfilling the selection criteria at these two days were included in the study until the required sample size was reached.

### Data collection

#### Pre-intervention assessment phase

1. A predesigned interviewing questionnaire was used at the beginning of the study to collect personal data, medical history, weight history, physical activity, and dietary habits.
2. Twenty-four-hour dietary recall technique was carried out as a dietary assessment tool.
3. Anthropometric measurements, height (Ht), weight (Wt), waist circumference (WC), and hip circumference (HC), were measured [17]. Body mass index (BMI) was calculated as weight (kg)/height (m)^2^. Waist-hip ratio (WHR) was calculated by dividing the waist circumference by the hip circumference. Waist-height ratio (WHtR) was calculated by dividing the waist circumference by the measured height in centimeters.
4. Assessment of total fat mass, skeletal muscle mass, and total body water was done using InBody720 which depends on the bioelectrical impedance analysis (BIA) technique [18], then body fat percentage, skeletal muscle percentage, and body water percentage were calculated.
5. Laboratory investigations were carried out where blood samples were collected in heparinized syringes after 8 h overnight fast. Fasting serum glucose [19] and fasting serum insulin levels [20] were determined. Quantitative determination of the insulin level was performed using an enzyme-linked immunosorbent assay (ELISA) kit supplied from DRG instruments GmbH, Germany (EIA-2935). The degree of insulin resistance was calculated by the updated computer homeostasis model assessment (HOMA2) [21] and referred to as homeostasis model assessment for insulin resistance (HOMA-IR). Serum samples were stored at − 20^o^C unit until serum total adiponectin concentration was measured using commercial ELISA (human adiponectin ELISA kit; Boster Immunoleader, Pleasanton, CA, catalog number EK0595) [22].

#### Intervention phase

Weight loss program: All subjects were instructed to eat a well-balanced diet aiming at reducing 500–800 kcal/day less than individually calculated energy requirements, with the goal to achieve a rate of weight loss of 0.5–1.0 kg/week [23]. Resting energy expenditure (REE) was calculated by the Mifflin St Jeor equation [24], then it was multiplied by a coefficient of correction for physical activity level to get energy requirements. The diet was designed to provide 20–35% of energy derived from fat, 45–65% from carbohydrates, and 10–35% from proteins [25]. Subjects were advised to exercise or walk 30 min per day, 5 days a week.

#### Follow-up phase

Dietary instructions were reinforced and monitored weekly throughout the 16-week intervention period. Compliance to physical activity was recorded weekly. The females were classified according to their compliance to physical exercise recommendation into three categories: no physical exercise, irregular physical exercise, and physical exercise as recommended.

#### Evaluation phase

At the end of the 16-week intervention period, the outcomes of the intervention were evaluated through reassessment of changes in the anthropometric measurements, body components, and laboratory investigations.

### Ethical consideration

Approval of the Ethics Committee of HIPH, Alexandria University, Egypt, was obtained on 9 February 2016. All patients were informed, and a written consent was obtained from all participants after explaining the aim of the study.

### Data management and statistical analysis

Data were managed and analyzed using the statistical software IBM SPSS version 20. All statistical analyses were done using a two-tailed test and alpha error 0.05. Numeric data was tested for normality using the Kolmogorov-Smirnov test. Normally distributed data were presented as mean ± standard deviation (SD). Student’s *t* test, one-way analysis of variance (ANOVA), paired *t* test, simple correlation coefficient, and multiple linear regression were used in analyzing data [26].

## Results

The mean age of the sample was 31.12 ± 7.18 years. The anthropometric and laboratory characteristics of the studied sample before and after the intervention are presented in Table 1. After the intervention, body weight and BMI decreased significantly by 9.7% (*P* < 0.0001) and also WC decreased significantly by 8.2% (*P* < 0.0001). WHR and WHtR decreased significantly by 2.7% and 8.2%, respectively (*P* < 0.0001). The changes in the body composition with the interventions indicated a significant decrease in the percent of body fat by 7.8% (*P* < 0.0001) and a significant increase in the percent of body muscle and body water by 6% and 6.9%, respectively (*P* < 0.0001). The laboratory changes after the intervention demonstrated a significant increase in adiponectin by 50.2% (*P* < 0.0001) from 13.3 ± 4.9 μg/ml to 18.5 ± 5.6 μg/ml. Both fasting insulin and HOMA-IR had decreased significantly by 13.6% and 13.7%, respectively (*P* < 0.0001), but there was a slight insignificant decrease in fasting blood glucose (0.2%).

**Table 1:** Anthropometric and laboratory characteristics of the sample of overweight and obese women before and after the intervention (*n* = 95), Alexandria, Egypt, 2016-2017

Characteristics	Before, mean ± SD	After, mean ± SD	Percent of change (%)	***t***_***d***_	***P*** value
Weight, kg	94.4 ± 18.5	85.1 ± 16.9	− 9.7	18.185	< 0.001*
Body mass index, kg/m^2^	35.8 ± 6.9	32.3 ± 6.2	− 9.7	18.118	< 0.001*
Waist circumference, cm	100.9 ± 12.4	92.6 ± 11.5	− 8.2	17.425	< 0.001*
Waist-hip ratio (WHR)	1.03 ± 0.07	1.00 ± 0.08	− 2.7	5.056	< 0.001*
Waist-height ratio (WHtR)	0.62 ± 0.079	0.57 ± 0.075	− 8.2	17.609	< 0.001*
Body fat percent, %	47.4 ± 4.4	43.7 ± 5.4	− 7.8	12.837	< 0.001*
Body muscle percent, %	29.0 ± 2.3	30.7 ± 2.8	+ 6.0	9.742	< 0.001*
Body water percent, %	38.6 ± 3.1	41.2 ± 3.9	+ 6.9	13.124	< 0.001*
Serum total adiponectin (μg/ml)	13.3 ± 4.9	18.5 ± 5.6	+ 50.2	8.701	< 0.001*
Fasting serum glucose (mg/dl)	89.9 ± 12.1	88.8 ± 12.1	− 0.22	0.813	0.418
Fasting serum insulin (μIU/ml)	10.3 ± 5.6	7.7 ± 3.3	− 13.6	6.376	< 0.001*
HOMA-IR	1.3 ± 0.7	1.0 ± 0.4	− 13.7	6.338	< 0.001*

SI conversion factors: to convert glucose from mg/dl to mmol/l, multiply by 0.0555; and insulin from μIU/ml to pmol/l, multiply by 7.175

Abbreviations: *t*_*d*_ *t* Difference in paired *t* test, *HOMA-IR* Homeostasis model assessment for insulin resistance

*Statistically significant

**Fig. 1:**
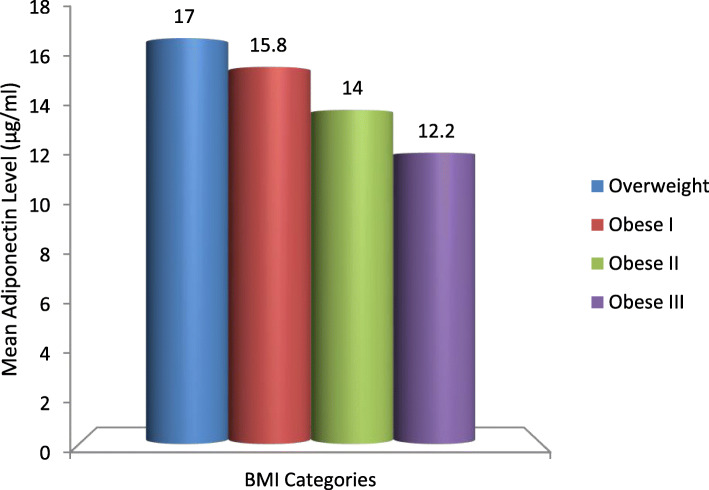
The mean adiponectin level among the studied sample according to BMI categories

An inverse relationship between the mean of the adiponectin level and BMI was observed. The mean adiponectin level among females with overweight was higher than the level of those with grade III obesity (17.0 μg/ml and 12.2 μg/ml, respectively), and also the mean adiponectin level among females with grade I obesity (15.8 μg/ml) was higher than the level of those with grade III obesity, and both differences were statistically significant (*F* = 3.817, *P* = 0.013) (Fig. 1).

The correlation coefficients of total adiponectin, HOMA-IR before the intervention, and some anthropometric and laboratory characteristics are shown in Table 2. There was a significant direct correlation between adiponectin level and both body muscle percentage (*r* = 0.302) and body water percentage (*r* = 0.317), while there was a significant indirect correlation between adiponectin level and each of WHR (*r* = − 0.393), body fat percentage (*r* = − 0.308), body weight (*r* = − 0.204), fasting insulin (*r* = − 0.210), and HOMA-IR (*r* = − 0.195). On the other hand, there was a significant direct correlation between HOMA-IR and each of body weight (*r* = 0.474), BMI (*r* = 0.435), WC (*r* = 0.330), WHtR (*r* = 0.278), and body fat percentage (*r* = 0.226), while there was a significant indirect correlation between HOMA-IR and body water percentage (*r* = − 0.226).

**Table 2:** Correlations of anthropometric and laboratory characteristics with serum total adiponectin and HOMA-IR before the intervention (*n* = 95)

Characteristics	Serum total adiponectin	*P* value	HOMA-IR	*P* value
Body weight	− 0.204^a^	0.047	0.474^a^	< 0.01
Body mass index	− 0.119	0.249	0.435^a^	< 0.01
Waist circumference	− 0.103	0.318	0.330^a^	< 0.01
Waist-hip ratio (WHR)	− 0.393^a^	0.001	0.186	0.071
Waist-height ratio (WHtR)	− 0.033	0.751	0.278^a^	0.006
Body fat percentage	− 0.308^a^	0.002	0.226^a^	0.028
Body muscle percentage	0.302^a^	0.003	− 0.147	0.155
Body water percentage	0.317^a^	0.002	− 0.226^a^	0.028
Fasting serum glucose	0.170	0.099	0.393^a^	< 0.01
Fasting serum insulin	− 0.210^a^	0.041	0.998^a^	< 0.01
HOMA-IR	− 0.195^a^	0.050	N/A	–
Serum total adiponectin	N/A	–	− 0.195^a^	0.050

*Abbreviations*: *HOMA-IR* Homeostasis model assessment for insulin resistance, *N/A* Not applicable

^a^Statistically significant

Two multiple regression analysis models (multivariable) to uncover factors which might be related to adiponectin level and HOMA-IR among the studied sample before intervention were done (Tables 3 and 4). WHR (*B* = − 22.836, SE *B* = 6.687, *P* = 0.001), body fat percentage (*B* = − 0.343, SE *B* = 0.152, *P* = 0.027), and HOMA-IR (*B* = − 1.545, SE *B* = 0.714, *P* = 0.033) were independently, negatively, and significantly associated with serum total adiponectin level [the unstandardized beta (*B*), the standard error for the unstandardized beta (SE *B*), and the probability value (*P*)] (Table 3), while only BMI (*B* = 0.029, SE *B* = 0.010, *P* = 0.003) was independently, positively, and significantly associated with HOMA-IR (Table 4).

**Table 3:** Multiple regression analysis of variables associated with adiponectin level before the intervention

Factor	***B***	SE	Beta	***t***	Sig.
History of practicing physical activity	0.552	0.165	0.294	3.341	< 0.01*
Number of main meals	0.934	0.418	0.207	2.232	0.028*
Body mass index	− 0.259	0.097	0.366	− 2.668	0.009*
Waist-hip ratio	− 22.836	6.687	− 0.339	− 3.415	< 0.01*
Percent body fat	− 0.343	0.152	− 0.307	− 2.254	0.027*
HOMA-IR	− 1.545	0.714	− 0.229	− 2.163	0.033*
*R*^2^ = 36.1% *F* = 7.017, *P* = 0.0001*					

*Statistically significant

**Table 4:** Multiple regression analysis of variables associated with HOMA-IR before the intervention

Factor	***B***	SE	Beta	***t***	Sig.
Family history of obesity	0.399	0.161	0.245	2.478	0.015*
Number of main meals	0.187	0.070	0.257	2.669	0.009*
Body mass index	0.029	0.010	0.280	3.036	0.003*
*R*^2^ = 44.0% *F* = 12.582, *P* = 0.0001*					

*Statistically significant

The correlation coefficients between differences in each of adiponectin level, HOMA-IR, and some anthropometric and laboratory characteristics of the sample are shown in Table 5. There was no statistically significant correlation between the increment in adiponectin level and the change in any anthropometric or laboratory parameters. A significant direct correlation between the difference in HOMA-IR and difference in each of WHR (*r* = 0.310), body weight (*r* = 0.238), BMI (*r* = 0.229), and WC (*r* = 0.202) was found.

**Table 5:** Correlations of anthropometric and laboratory characteristics with change (after-before) in serum total adiponectin and HOMA-IR (*n* = 95)

	Change in serum total adiponectin	***P*** value	Change in HOMA-IR	***P*** value
Change in body weight	− 0.106	0.304	0.238^a^	0.020
Change in body mass index	− 0.095	0.362	0.229^a^	0.026
Change in waist circumference	− 0.099	0.342	0.202^a^	0.049
Change in waist-hip ratio	− 0.020	0.847	0.310^a^	0.002
Change in waist-height ratio	− 0.095	0.357	0.200	0.052
Change in body fat percentage	− 0.003	0.975	0.129	0.214
Change in fasting serum glucose	0.068	0.511	0.187	0.070
Change in fasting serum insulin	− 0.081	0.433	0.996^a^	< 0.001
Change in HOMA-IR	− 0.093	0.372	N/A	–
Change in serum total adiponectin	N/A	–	− 0.093	0.372

*Abbreviations*: *HOMA-IR* Homeostasis model assessment for insulin resistance, *N/A* Not applicable

^a^Statistically significant

A multiple regression analysis to uncover factors which might be related to the change in HOMA-IR was done. The model explained 22.3% of the variation in the difference of HOMA-IR, and it was statistically significant (*F* = 5.103, *P* = 0.0001). Body mass index (*B* = 0.095, SE *B* = 0.029, *P* = 0.002) and WHR (*B* = 2.746, SE *B* = 0.989, *P* = 0.007) were independently, positively, and significantly associated with the change in HOMA-IR (Table 6).

**Table 6:** Multiple regression analysis of variables associated with change (after-before) in HOMA-IR

Factor	***B***	SE	Beta	***t***	Sig.
Difference in BMI	0.095	0.029	0.352	3.224	0.002*
Difference in waist-hip ratio	2.746	0.989	0.297	2.777	0.007*
*R*^2^ = 22.3% *F* = 5.103, *P* = 0.0001*					

*Statistically significant

Patients were classified according to compliance to physical activity recommendations into three categories as shown in Table 7. There was no statistically significant difference between the mean differences of any anthropometric measurements, adiponectin level, or insulin resistance among the three categories of the sample.

**Table 7:** Mean difference of anthropometric and laboratory characteristics before and after the intervention according to compliance with physical exercise

Mean difference in the variables (after-before) intervention	No exercise, *n* = 41	Irregular exercise, *n* = 44	Regular exercise, *n* = 10	Sig. *F*	*P* value
Weight (kg)	− 9.48	− 9.59	− 7.23	0.963	0.385
Body mass index, kg/m^2^	− 3.58	− 3.65	− 2.75	0.947	0.392
Waist circumference, cm	− 8.32	− 8.66	− 6.90	0.576	0.564
Waist-hip ratio (WHR)	− 0.032	− 0.026	− 0.029	0.096	0.908
Waist-height ratio (WHtR)	− 0.051	− 0.053	− 0.043	0.565	0.570
Body fat percentage	− 3.27	− 4.20	− 2.77	1.786	0.173
Body muscle percentage	1.44	2.04	1.33	1.602	0.207
Body water percentage	2.39	3.06	2.03	1.817	0.168
Serum total adiponectin (μg/ml)	5.34	5.03	5.54	0.046	0.955
Fasting serum glucose (mg/dl)	− 1.59	− 0.89	0.00	0.068	0.935
Fasting serum insulin (μIU/ml)	− 3.19	− 2.36	− 1.35	1.013	0.367
HOMA-IR	− 0.41	− 0.30	− 0.18	1.021	0.364

*Abbreviations*: *F* variation between sample means/variation within the samples in the ANOVA test, *HOMA-IR* Homeostasis model assessment for insulin resistance*, N/A* Not applicable

## Discussion

In this study, the hypothesis that a short-term (16 weeks) weight reduction program depending mainly on low caloric balanced diet can change serum total adiponectin level and insulin resistance was tested. In contrast to other adipokines and, although it is produced by adipocytes, adiponectin level is paradoxically lower in obese subjects than in non-obese subjects as revealed in a previous study [8]. This finding suggests that adipose tissue may exert a negative feedback on adiponectin production. In the present study, the mean total plasma adiponectin level was slightly higher than expected hypoadiponectinemia associated with overweight and obesity. This finding may be due to the wide range of normal reference (3–30 μg/ml) [5].

Results concerning changes in adiponectin level after weight loss in many studies are inconsistent. Several studies demonstrated no change in total plasma adiponectin after a short-term weight reduction intervention despite a significant reduction in body weight [27–29]. On the other hand, large weight reduction following bariatric surgery was accompanied by an increase in plasma adiponectin level [30, 31]. It has been demonstrated from the present study that a moderate weight loss of 9.3 kg (represented 9.7% of the original weight) induced by a balanced low caloric diet was accompanied by a significant increase in serum total adiponectin level from 13.3 to 18.5 μg/ml, which represented 50.2% increase from the original level and a decrease in insulin resistance by 13.7%.

The change in adiponectin level was not significantly correlated with the change in insulin sensitivity or the improvement in obesity parameters; this could be explained by the short period of the intervention. Meanwhile, the decline in insulin resistance was positively correlated to the decline in body weight, BMI, WC, and WHR. Regression analysis revealed that difference in BMI and difference in WHR were the two predictors for the improvement in insulin resistance index.

The relation between adiponectin level and BMI is inconsistent. Many studies reported a negative association between them [32–34], while another study demonstrated no association [35]. The present study revealed no significant correlation between them. Previous studies reported a negative correlation between adiponectin level and waist circumference as a parameter for increased abdominal fat accumulation [36, 37]. In the current study, we investigated three parameters (WC, WHR, and WHtR) to indicate the relation between adiponectin level and abdominal obesity. Of these parameters, WHR was the most appropriate indicator, as it was negatively related to adiponectin level, and was one of the predictors of adiponectin level in the multiple regression model.

Apart from negative correlations with adiposity measures, studies revealed that adiponectin levels seem to be reduced prior to the development of type 2 diabetes, even after adjusting for measures of obesity, and that administration of adiponectin has been accompanied by increased insulin sensitivity [38, 39]. The present study extends findings of these studies by providing an example of an obesity-independent association of insulin resistance with adiponectin levels, as there was a significant indirect correlation between HOMA-IR and mean level of total adiponectin; also, a multiple regression analysis revealed HOMA-IR to be one of the significant independent predictors of total adiponectin level.

Regarding physical exercise as a part of the weight reduction program, a previous study reported that a weight loss program that included exercise increased the plasma adiponectin levels of obese women in a randomized trial [40]. In the present study, there was no statistically significant difference between the change in the mean adiponectin level or the mean of any anthropometric parameters, percent of body components, or insulin sensitivity among the three categories of the sample regarding physical activity compliance. This finding supports that the low caloric diet represented the more effective part of the intervention program; also, it raises the suggestion that more time is needed for the physical activity to affect the adiponectin level or cause a change in anthropometric measurements or insulin sensitivity.

### Limitations of the study

There were certain limitations in undertaking this study; first, the design adopted in the study was one arm design lacking the control limb. Although this study design is considered a weak one, for ethical considerations, it was unethically to take a group of obese patients as a control group without doing any intervention for them despite being aware of the health hazards of obesity. The duration of the intervention program was the second limitation, after analyzing data; the need for more than 16 weeks of intervention was raised to test the effect of exercise on adiponectin level. This duration was chosen to decrease the number of dropouts and the expectation of dropouts was 10%, but actually, they reached 22%.

## Conclusion

Lifestyle-related factors, such as overeating and physical inactivity, induce the accumulation of visceral fat which may lead to dysfunction of adipocytes. Hyposecretion of defensive adiponectin might represent obvious mechanisms of lifestyle-related diseases, including diabetes mellitus, hypertension, hyperlipidemia, and atherosclerosis. Reduction of visceral fat is recommended as it can be a major preventive measure for the metabolic syndrome and its consequences, as our study revealed that a moderate weight loss following a balanced low caloric diet was associated with a significant increase in serum total adiponectin level, percent of muscle mass and body water, and a significant reduction in insulin resistance, and waist circumference.

## Data Availability

The datasets used and analyzed during the current study are available from the corresponding author on reasonable request.

## Abbreviations

ANOVA: Analysis of variance
BIA: Bioelectrical impedance analysis
BMI: Body mass index
ELISA: Enzyme-linked immunosorbent assay
HC: Hip circumference
HIPH: High Institute of Public Health
Ht: Height
HOMA: Homeostasis model assessment
HOMA-IR: Homeostasis model assessment-insulin resistance
IL-6: Interleukin-6
IR: Insulin resistance
*n*: Number
*P*: *P* value
REE: Resting energy expenditure
SD: Standard deviation
Sig.: Significance
SPSS: Statistical Package of Social Science
TNF-α: Tumor necrosis factor-α
WC: Waist circumference
WHR: Waist-hip ratio
WHtR: Waist-height ratio
Wt: Weight
